# A better-ventilated ocean triggered by Late Cretaceous changes in continental configuration

**DOI:** 10.1038/ncomms10316

**Published:** 2016-01-18

**Authors:** Yannick Donnadieu, Emmanuelle Pucéat, Mathieu Moiroud, François Guillocheau, Jean- François Deconinck

**Affiliations:** 1 Laboratoire des Sciences du Climat et de l'Environnement, LSCE-IPSL, CEA-CNRS-UVSQ, Université Paris-Saclay, 91191 Gif sur Yvette, France; 2 Biogéosciences Dijon, Université Bourgogne-Franche-Comté, UMR CNRS 6282, Dijon 21000, France; 3 Géosciences Rennes, Université de Rennes, UMR CNRS 6118, Rennes 35042, France

## Abstract

Oceanic anoxic events (OAEs) are large-scale events of oxygen depletion in the deep ocean that happened during pre-Cenozoic periods of extreme warmth. Here, to assess the role of major continental configuration changes occurring during the Late Cretaceous on oceanic circulation modes, which in turn influence the oxygenation level of the deep ocean, we use a coupled ocean atmosphere climate model. We simulate ocean dynamics during two different time slices and compare these with existing neodymium isotope data (*ɛ*_Nd_). Although deep-water production in the North Pacific is continuous, the simulations at 94 and 71 Ma show a shift in southern deep-water production sites from South Pacific to South Atlantic and Indian Ocean locations. Our modelling results support the hypothesis that an intensification of southern Atlantic deep-water production and a reversal of deep-water fluxes through the Caribbean Seaway were the main causes of the decrease in *ɛ*_Nd_ values recorded in the Atlantic and Indian deep waters during the Late Cretaceous.

## Abstract (web summary)

In the Earth's history, the timing of oceanic large-scale events of oxygen depletion remains poorly understood. Here, the authors show that palaeogeography was a major preconditioning factor during the Cretaceous, implying that thresholds to shift toward a global anoxia are likely to be much higher at present.

## Graphical abstract

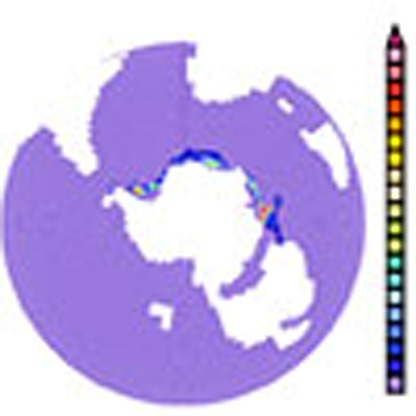

In the context of recent warming, modern ocean de-oxygenation that occurs not only on continental margins but also in the tropical oceans worldwide^1^ resembles the model initially invoked for the onset of oceanic anoxic events (OAEs)^2^. Recent studies point to a major role of increased nutrient inputs as a trigger for OAEs^3^,^4^,^5^. Nevertherless, ocean circulation, through its impact on oxygen concentration in deep waters, may have affected the thresholds required to trigger an OAE. It has been suggested that Late Cretaceous changes in climate and continental configuration^6^,^7^,^8^, namely the widening of the Atlantic Ocean and the deepening of the Central Atlantic (CA) gateway^9^, could have induced major changes in oceanic circulation that may have had an impact on the general oxygenation state of the oceanic basins and contributed to the conclusion of these large-scale anoxic events in the deep ocean^10^,^11^.

Nevertheless, no consensus exists on oceanic circulation modes and their possible evolution during the Cretaceous, despite recent improvements of the spatial and temporal coverage of neodymium isotopic data (ɛ_Nd_), a proxy for oceanic circulation^11^,^12^,^13^,^14^,^15^. For instance, a decrease in bottom water ɛ_Nd_ values during the Late Cretaceous in the Atlantic and Southern Oceans has been interpreted either as reflecting the initiation or intensification of deep-water production in the northern Atlantic^15^ or in the South Atlantic (SA) and in the Indian Ocean^10^,^11^,^13^. Additional sources for deep waters have been suggested in the North or South Pacific, or even at low latitudes, based on ɛ_Nd_ or oxygen isotope data^16^,^17^,^18^.

General circulation models have also been used to study oceanic circulation during the Cretaceous^7^,^19^,^20^. To our knowledge, no modelling studies have reconstructed the evolution of ocean dynamics resulting from the widening of the South and CA Ocean occurring between the Cenomanian and the Maastrichtian. Published simulations either focus on a specific time period^20^,^21^ or are devoted to the impact of the CA gateway opening between the Albian and the Cenomanian^22^.

Here we use the fast ocean atmosphere model (FOAM), to explore the evolution of oceanic circulation occurring during the Late Cretaceous (see Methods). Our simulations highlight an evolution from a sluggish circulation in the South and CA using a Cenomanian/Turonian land–sea mask towards a much more active circulation in these basins with an early Maastrichtian land-sea mask.

## Results

### Changes in oceanic circulation at 1,120 p.p.m

**Figure 1:**
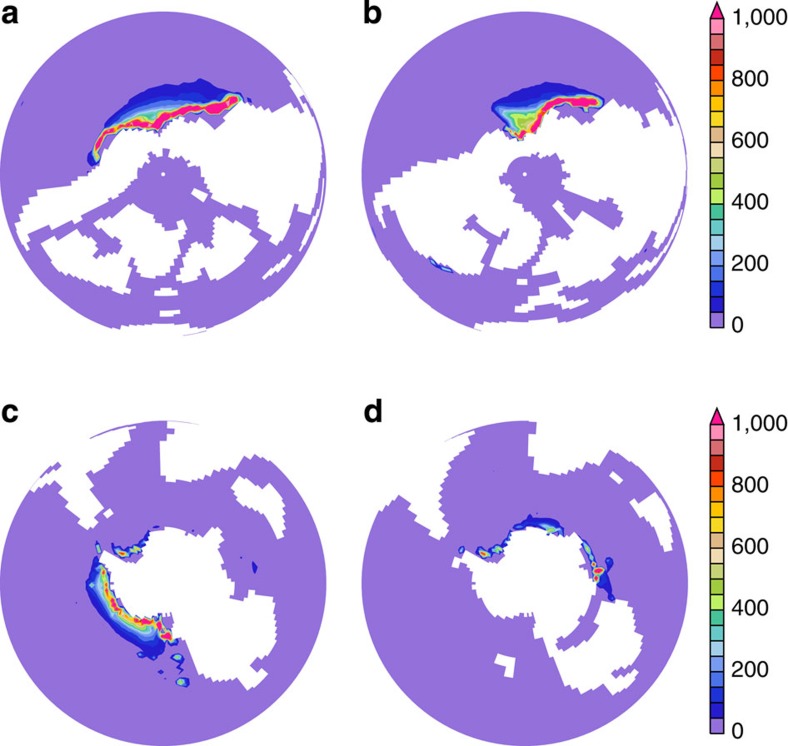
Annual distribution of convective adjustments across the water column. (**a**) For 94 Ma, 4 × CO_2_ from the North Pole. (**b**) For 71 Ma, 4 × CO_2_ from the North Pole. (**c**) For 94 Ma, 4 × CO_2_ from the South Pole. (**d**) For 71 Ma, 4 × CO_2_ from the South Pole. The shading signifies the number of times the water column has undergone convective mixing summed over a year. Regions experiencing a large occurrence of convective adjustments are interpreted to represent site of intermediate and deep-water formation.

The 94 Ma simulation at a CO_2_ concentration of 1,120 p.p.m. (four times the pre-industrial atmospheric level) displays a bipolar oceanic circulation characterized by large areas of deep-water formation located both in the north and in the south of the Pacific Ocean (Fig. 1). The production of deep waters along the northwest boundary of the Pacific Ocean is common to many numerical Cretaceous simulations^4^,^20^,^22^ but not all^23^. The sinking of water masses occurs over the cyclonic subpolar gyres and results from the winter cooling of warm and salty surface waters located within this large region^20^. The use of the early Maastrichtian land–sea mask for the same atmospheric CO_2_ level induces substantial changes in the location of deep-water production in the model. The north Pacific area of deep-water formation is reduced to the northwestern Pacific area only and sinking in the south Pacific area completely disappears. Conversely, the Atlantic and the Indian sectors of the Austral Ocean become larger contributors of deep waters and a small area of intermediate-water formation appears along the northeast American margin in the North Atlantic (Fig. 1).

### Sensitivity to atmospheric CO_2_ levels

Changes in atmospheric CO_2_ level from 8 to 2 pre-industrial atmospheric level have a small effect on the distribution of deep-water formation areas in our study (Supplementary Figs 1 and 2). The main features of oceanic circulation modes remain quite similar, with a disappearance of deep-water convection in the southern Pacific and a larger area of deep-water formation appearing in the southern Atlantic when using early Maastrichtian palaeogeography.

Nevertheless, the sensitivity of overturning to CO_2_ level remains an open debate, as some models^24^,^25^ found that ocean dynamics vary with CO_2_ while others did not^4^,^26^. We emphasize that all simulations presented here represent an equilibrated climate. The ocean can go through a transient stratified state resulting from a faster warming of the oceanic upper layers when confronted with a CO_2_ increase imposed on a short time scale (a few hundred to a few thousand years). Winter convection at high latitudes would then stop, owing to the decrease in vertical density gradient, and the classical thermohaline circulation may stop as well, but only transiently. We thus recognize that there is room for improvement in this area of research and await a modelling inter-comparison project for warm climates such as the one that has been initiated on the warm Eocene climate^27^; however, such a comparison is beyond the scope of this study.

The results of our model suggest at this point that the changes in ocean dynamics simulated between 94 and 71 Ma are driven by changes in palaeogeography rather than by the cooling recorded between the Cenomanian and the Maastrichtian. As only minor changes occur with varying CO_2_ levels, only the simulations at 1,120 p.p.m. will be further described and compared for each palaeogeography in the remainder of the study. Our main aim in this study is to describe and understand the changes in ocean dynamics that occur. It is however interesting to note that the atmospheric CO_2_ concentration range used in our simulations produces sea-surface and deep-water temperatures that are comparable to oceanic latitudinal thermal gradients reconstructed from palaeoceanographic data for the Cenomanian/Turonian boundary and for the early Maastrichtian (Supplementary Fig. 7).

### Pathways of water masses between the oceanic basins

**Figure 2:**
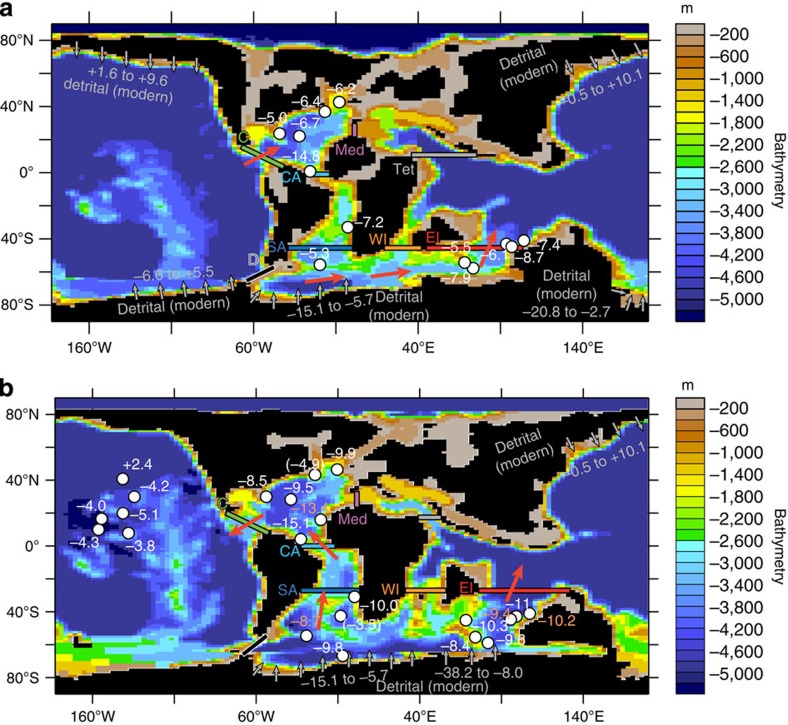
Land–sea distribution and bathymetry specified in the FOAM simulations. (**a**) Cenomanian/Turonian boundary (94 Ma). (**b**) Early Maastrichtian (71 Ma). Latitudinal and meridional sections across which water transport has been calculated for Fig. 3 are shown. C, Caribbean Seaway; CA, Central Atlantic; D, Drake Passage; EI, East India; Med, Mediterranean; SA, South Atlantic; Tet, Tethys; WI, West India. Supplementary Figs 3 and 4 show the latitude/longitude—depth profile for each section except for the c and d sections for which the bathymetry of the area of interest is shown. The red arrows schematically show the deep-water pathways deduced from our modelling experiments. *ɛ*_Nd_(*t*) values of intermediate and deep waters (in white) inferred from fish remains and oxide coatings and averaged for the Turonian (**a**) and Maastrichtian (**b**) are reported at available ODP sites (see Supplementary Fig. 5 for the names of each ODP site). Details on the calculation of average *ɛ*_Nd_(*t*) values can be found in Supplementary Data 2. For ODP sites at Demerara Rise (Central Atlantic), *ɛ*_Nd_(t) values within OAE2 have not been included in the calculation. Values in parentheses indicate a contamination of the samples or a contamination of deep waters by volcanogenic particles^11^,^12^. Values in orange on the Maastrichtian map (**b**) are from the late Campanian. *ɛ*_Nd_ values of the modern detrital fraction (core-top detrital fraction and continental lithogenic sources) in the regions of modelled deep-water production are reported in grey for discussion (details on the specified range of values are available in Supplementary Data 1 and 2, and Supplementary Figs 5 and 6 with associated references).

**Figure 3:**
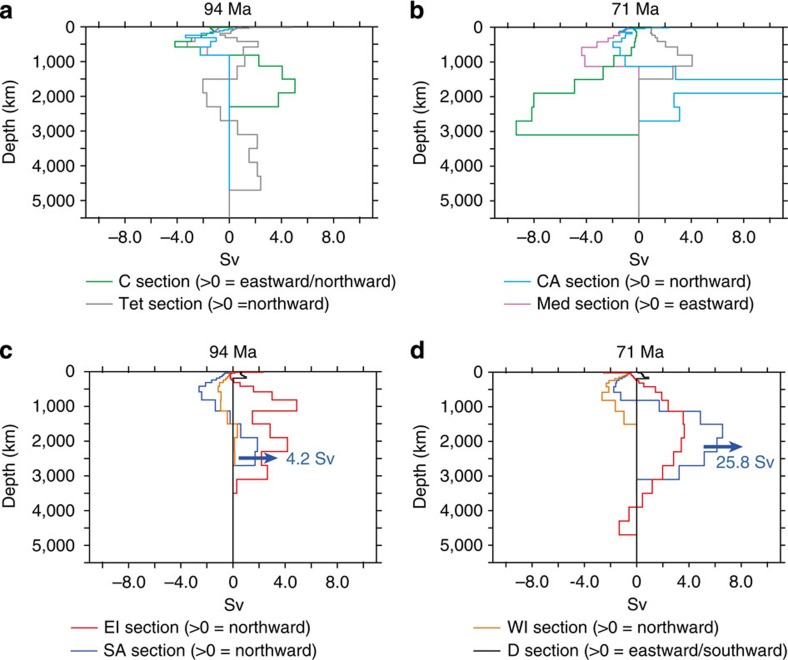
Water transports. As computed for the segments defined in Fig. 2 for the North Atlantic and the Tethys basins (**a**,**b**) and for the SA and Indian basins (**c**,**d**). The units are in Sv, that is, 10^6^ m^3^ s^−1^. Sv is used to quantify the volumetric rate of transport of ocean currents. Values corresponding to the water flow for each vertical level of the ocean model are plotted. The variable corresponds to the integration of the velocity V (U) on the *x* axis (or *y* axis depending on the orientation of the section) and on the *z* axis. Calculated water fluxes account for both the velocity and the area of the section at a given level of the model. For zonal water transports such as the one calculated for the Med segment, positive values indicate eastward transport. For meridional water transports such as those calculated for the Tethyan (Tet), Central Atlantic (CA), South Atlantic (SA), East Indian (EI) and West Indian (WI) segments, positive values indicate northward transport; negative values indicate southward transport. For the specific case of the D section, positive directions are Southward and Eastward, and for the **c** section, positive directions are Northward and Eastward. Blue arrows in **c**,**d** represent water fluxes computed across the SA section for the deeper vertical levels where the transport is northward.

To go a step beyond this first-order description of ocean dynamics, we have computed zonal and/or meridional water transport across several key sections of the ocean basins (Figs 2 and 3). In most previous modelling studies, ocean dynamics have been described through a global overturning function. However, a global overturning function may be dominated by the largest oceanic basin, that is, the Pacific Ocean, and does not allow for the identification of ocean dynamics occurring in the still relatively narrow Atlantic Ocean, in the Tethys or in the Indian Ocean. Our aim here is to provide a better three-dimensional picture of water flow within and between the different oceanic basins.

In the CTRL94Ma run, 7 Sv are transported from the South Pacific to the SA across the D section (Fig. 3c and Table 1). The circulation simulated within the SA Ocean appears quite sluggish at depth, with a very limited northward flow of 4.2 Sv throughout the SA section between 1,200 and 2,750 m (Fig. 3c and Table 1). Conversely, across the East India section, water flow is directed northward down to a depth of 3,000 m and transports 25 Sv to the North (Fig. 3c and Table 1). In the CTRL71Ma run, deep-water production intensifies in the southern Atlantic, extends eastward to the Indian sector and does not occur in the southern Pacific sector any more (Figs 1 and 3). Figure 3d shows a drastic intensification of the northern flow across the SA section for depth below 800 m. These northward-flowing deep waters reach the North Atlantic, as testified by the large flux of water across the CA section (Fig. 3b). Surface-to-intermediate waters across the Central to the Southern Atlantic are also flowing more intensively from the North to the South.

In the CTRL94Ma run and in the North Atlantic, the vertical flux profiles computed for the Caribbean Seaway section, CA section and Mediterranean (Med) section indicate that the surface and intermediate waters down to about 1,000 m flow from the Tethys to the North Atlantic, and then southward across the CA Gateway and westward into the equatorial Pacific via the Caribbean Seaway. Across the Caribbean Seaway below 1,000 m, waters flow from the Pacific to the Atlantic as shown by the eastward water flux from 1,000 to ∼2,500 m (Fig. 3). The existence of an estuarine circulation pattern, with the surface layers flowing from the Tethys to the Pacific and the deeper layers flowing in the opposite direction, has been noticed several times in previous modelling studies^4^,^19^,^21^. In the CTRL71Ma runs, the model simulates an increase in circum-equatorial transport across the Caribbean Seaway and the Med gateway. Deep waters are now flowing from the Atlantic to the Pacific at all depths across the Caribbean Seaway, which marks the end of estuarine circulation in the Atlantic.

### Comparison with available *ɛ*_Nd_

Nd in seawater ultimately derives from continents and the rocks eroded around an area of deep-water production imprint the surface waters with a distinct isotopic composition that depends on the age and lithology of the rock^28^. This signature is then exported to depth as the water sinks. The residence time of Nd in the ocean is shorter than the oceanic mixing time, which prevents complete homogenization but is long enough for Nd to be transported by deep-water masses along their pathways^28^. The Nd isotope composition of deep waters has thus been used to track oceanic circulation patterns in both modern and ancient oceans^10^,^28^,^29^,^30^.

Available data for the Late Cretaceous highlight a major decrease in deep-water *ɛ*_Nd_ recorded both in the North and South Atlantic and in the Indian Ocean^10^,^14^,^31^, with more unradiogenic (lower) *ɛ*_Nd_ values recorded during the Maastrichtian (on average in the −8.5 to −11 *ɛ*-units range) compared with the Turonian (on average in the −5 to −8.5 *ɛ*-units; Fig. 2). This decrease has been interpreted to reflect initiation of deep-water production in an area receiving unradiogenic Nd inputs from the nearby continents, with the northern Atlantic^15^, or the Atlantic or Indian sector of the Southern Ocean proposed as possible sources^10^,^11^. Indeed at present, *ɛ*_Nd_ values as low as −14 to −26 *ɛ*-units recorded in Baffin Bay and Labrador Sea waters reflect the unradiogenic Nd continental supply from nearby Archean terranes^32^,^33^,^34^ that were already present during the Cretaceous. In the southern Ocean, unradiogenic detrital inputs occur at present (Fig. 2, Supplementary Fig. 5 and Supplementary Data 1). The presence of similar unradiogenic inputs during the Late Cretaceous is supported by Campanian–Maastrichtian *ɛ*_Nd_ values of detrital material of around −10 *ɛ*-units at ocean drilling program (ODP) site 690 (ref. 12). Because of these unradiogenic detrital inputs from the Antarctic continent, the more radiogenic *ɛ*_Nd_ values (higher) recorded in the SA and Indian deep waters during the mid-Cretaceous have been interpreted^10^ to reflect at that time additional inputs of radiogenic Nd from abundant volcanic dust in the context of a sluggish circulation within a restricted basin.

It has also been suggested that the subsidence of Rio Grande Rise and other large igneous provinces (for example, Kerguelen or the Madagascar Plateau) during the Late Cretaceous could have diminished such inputs of radiogenic Nd to the southern Atlantic and Indian surface waters, contributing to decrease the *ɛ*_Nd_ values of the deep waters sourced in this region^31^.

The oceanic circulation modelled here is in agreement with existing *ɛ*_Nd_ and part of the proposed scenarios. In the CTRL94Ma runs, the model simulates deep-water formation in the northern and southern Pacific. In the modern ocean, the southern Pacific receives inputs of quite radiogenic Nd eroded from the nearby Antarctic continent (Fig. 2, Supplementary Fig. 5 and Supplementary Data 1). ^40^Ar/^39^Ar ages of detrital hornblende grains from West Antarctica^35^ support the presence of intrusive and volcanic rocks during the Cretaceous (and as early as the Jurassic) that probably provided radiogenic material to the nearby surface waters^36^, imprinting the deep water produced there with a radiogenic signature. Deep water produced in the North Pacific region should also have a radiogenic composition, because weathering of radiogenic young circum-Pacific volcanic arcs linked to the subduction of the Kula and Farallon plates was already active during the mid-Cretaceous^37^. This is supported by the high *ɛ*_Nd_ values of neritic seawater inferred from Late Cretaceous fish remains recovered from the northwestern Pacific, (typically in the −3 to +1 *ɛ*-units range)^38^. In the North Atlantic, deep waters below 1,000 m are conveyed in our simulation from the equatorial eastern Pacific through the Caribbean Seaway and result from the mixing of radiogenic deep waters formed both in the North and in the South Pacific. Therefore, the ocean dynamics simulated by the model are expected to result in quite radiogenic *ɛ*_Nd_ values of deep waters in the North Atlantic, in agreement with existing *ɛ*_Nd_ (Fig. 2 and Supplementary Data 2).

In the CTRL94Ma run, the deep waters bathing the southern Atlantic and Indian Oceans originate in the area located near the modern Weddell Sea, a region that most probably received unradiogenic continental supply from Antarctica as discussed above (Fig. 2). It has been suggested^10^,^31^ that the southern Atlantic may have received during the mid-Cretaceous additional inputs of radiogenic Nd from subaerially exposed continental volcanic provinces, exposure of submarine Large Igneous Provinces and hot spots, and from associated volcanic activity. The sluggish circulation simulated in this work in the SA in the CTRL94Ma run (Fig. 3 and Table 1) would have favoured seawater-particle exchange processes with such radiogenic volcanic particles^10^, resulting in a quite radiogenic composition of SA and Indian deep waters as depicted in the available Nd data set (Fig. 2).

In the CTRL71Ma run, convection in the southern Pacific Ocean completely disappears and is reduced in the North Pacific to the northwest area, whereas convection in the SA intensifies and extends eastward to Australia (Figs 2 and 3). The development of sites of deep-water formation near areas of probable unradiogenic continental supply in the southern Atlantic is expected to result in a relatively unradiogenic composition of SA and Indian Ocean waters. This is in agreement with available *ɛ*_Nd_ that display quite negative *ɛ*_Nd_ values of deep waters during the latest Cretaceous in the southern Atlantic and Indian Oceans, in the range of about −9.5 to −10.5 *ɛ*-units at ODP site 690 for the Late Campanian–Maastrichtian interval^12^ and of −8.4 to −11 *ɛ*-units on average for the Maastrichtian at several Indian Ocean ODP sites^10^,^12^,^13^ (Fig. 2).

Following the simulated circulation patterns, this unradiogenic deep-water composition would then be exported to the North Atlantic through the deeper CA gateway. In addition, with the inversion of the deep-water flux through the Caribbean Seaway in the model, the radiogenic Pacific deep waters would no longer enter the North Atlantic. A westward flux of deep waters throughout the Caribbean Seaway during the Campanian and Maastrichtian is supported by *ɛ*_Nd_ values of the detrital fraction higher than that of the bottom waters at site 152 (Nicaragua Rise)^39^. The oceanic circulation simulated for the Maastrichtian should thus generate a much less radiogenic Nd isotope signature of North and SA deep waters, as well as in the Indian Ocean down to 3,000 m of depth, in agreement with *ɛ*_Nd_ data (Fig. 2).

Our work thus supports an intensification of deep-water production in the southern Atlantic and Indian Oceans along with a reversal of the deep-water flux through the Caribbean Seaway as a driver for the decrease in deep-water *ɛ*_Nd_ depicted during the Late Cretaceous. The subsidence of large igneous provinces such as the Kerguelen plateau could also have contributed to reducing the inputs of radiogenic Nd to the surface waters of the southern Atlantic and Indian Oceans^31^. As Nd is not incorporated as a tracer into our model, the impact of reduced inputs of radiogenic Nd linked to this mechanism on the decrease recorded by deep-water *ɛ*_Nd_ values cannot be further discussed here.

The simulated circulation during the Maastrichtian remains difficult to reconcile with the highly non-radiogenic values recorded at Cape Verde and Demerara Rise^14^,^16^ in the low-latitude Atlantic Ocean. It has been suggested that intermediate to deep waters could have been generated in the area of Demerara Rise (‘Demerara Rise Bottom Waters')^16^. Nevertheless, the Late Cretaceous *ɛ*_Nd_ from Demerara Rise stands in marked contrast to the data from other North Atlantic sites^11^. Demerara Rise bottom waters may have been restricted to intermediate depths, similar to Med outflow water, and thus would not have greatly influenced deep-water masses in the North Atlantic^11^. Our results show no evidence for water sinking in this area (Fig. 1). One way to reconcile the *ɛ*_Nd_ of Demerara Rise and Cape Verde with the modelled circulation could be to invoke the impact of boundary exchange processes that are known to occur along continental margins and can modify the Nd isotope composition of the local bottom waters^32^,^40^. Considering the proximity of the two sites to very old, unradiogenic terranes (Guyana and African Shields), such a process may have locally lowered the Nd isotopic composition of the water masses. The increase in Nd isotope values recorded at this site at the end of the Maastrichtian when they reach values similar to the other Atlantic sites may then reflect the progressive opening of the Demerara Rise region to the remaining of the Atlantic.

### Mechanisms driving the changes in oceanic circulation

**Figure 4:**
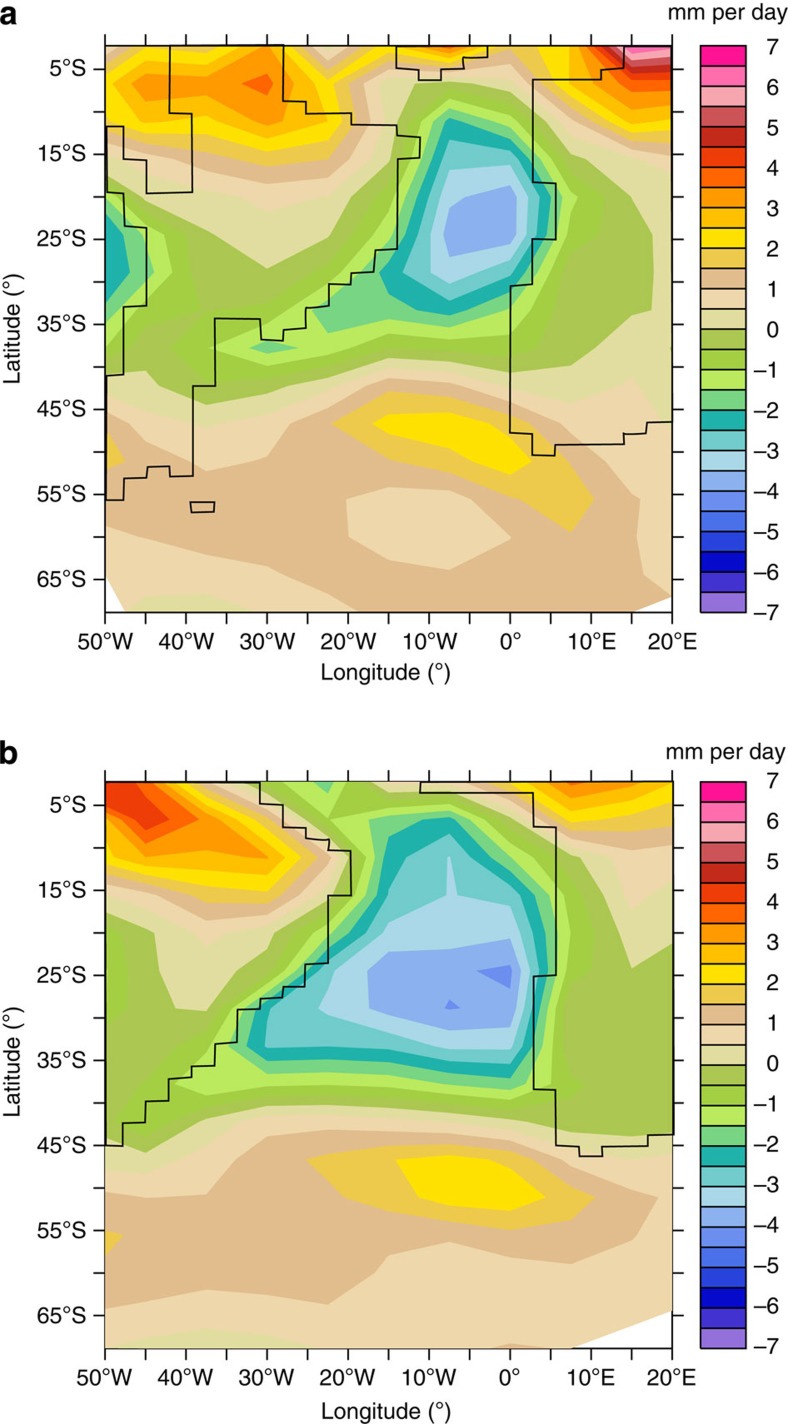
Hydrological budget over the Central and South Atlantic. Precipitation minus evaporation for 94 (**a**) and 71 Ma (**b**) runs.

**Figure 5:**
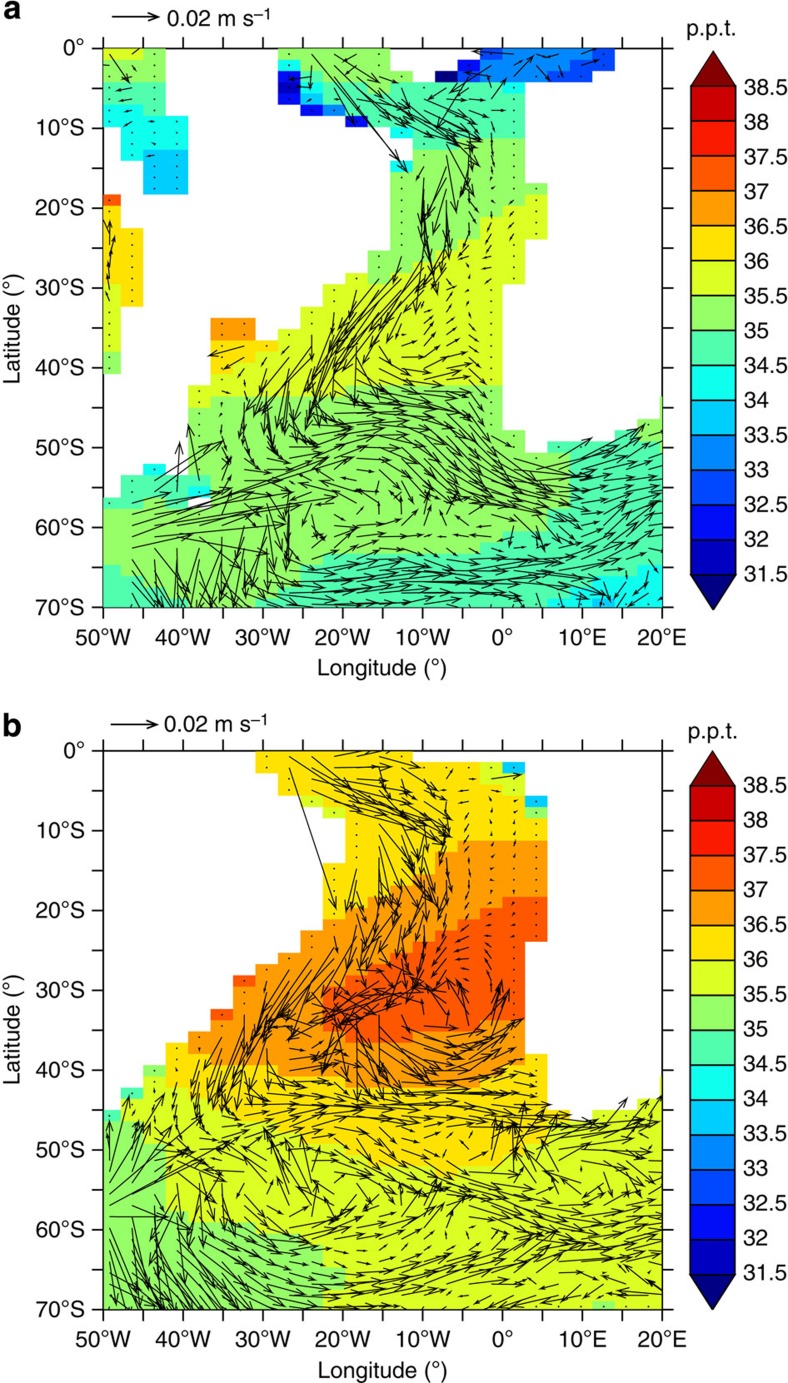
Absolute salinity fields. Salinity and current velocity (m s^−1^) averaged over the first 300 m of the ocean for 94 (**a**) and 71 Ma (**b**) runs.

In the model, the shift in the location of deep-water formation from the Pacific to the Atlantic/Indian sector is clearly due to modifications of the land–sea mask. In detail, it is the hydrological cycle over the SA basin that seems to be responsible for the shift in the deep-water formation area between the Cenomanian and the early Maastrichtian. During the early Maastrichtian, the fall in sea level and the retreat of the sea from the tropical continents induce more tropical monsoons and less vertical advection of moist air, which result in a more negative P−E (precipitation minus evaporation) budget in the CA Ocean (Fig. 4). When integrated over the latitudes 40°S–10°N, corresponding to the Central and SA, the P−E budget is more negative in the 71 Ma run with a value around—1.5 mm per day (−0.14 mm per day for the 94 Ma run). These changes will favour convection in the SA during the early Maastrichtian, because the saltier the surface waters are, the denser and heavier they will be when they reach the southern high latitudes (Fig. 5).

### Sensitivity to the Drake Passage and to the Caribbean Seaway

The results presented here are based on the two palaeogeographies published in Sewall *et al.*^9^, in which the depth of the Drake Passage has been reduced to 145 m. Major uncertainties exist on the configuration of the Drake Passage during the Late Cretaceous, with reported depths ranging from over 1,000 m^9^ to a shallow^41^ or completely closed passage^42^. Similarly, the depth and configuration of the Caribbean gateway is not well constrained. Island arc volcanism was already present across the Caribbean Seaway during the Late Cretaceous, possibly partly impeding deep or intermediate water communications across the seaway^43^,^44^. Nevertheless, the configuration of these two seaways have been shown to have important implications for oceanic circulation in more recent periods^45^,^46^.

**Figure 6:**
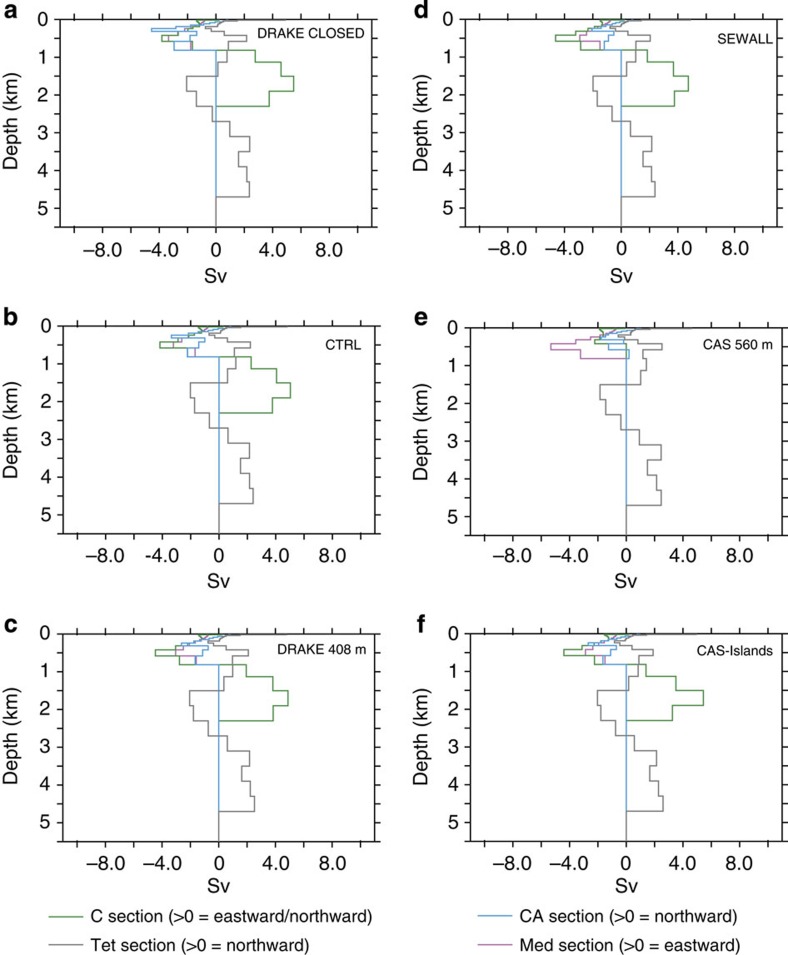
Water transports for the North Atlantic and the Tethys basins at 94 Ma. (**a**) Drake Passage closed (DRAKE CLOSED), (**b**) Drake Passage at 145 m (CTRL), (**c**) Drake Passage at 408 m (DRAKE408m), (**d**) Drake Passage as in Sewall *et al.*^9^ (SEWALL), (**e**) Caribbean gateway at 560 m (CAS560m) and (**f**) continental plate across the Caribbean gateway (CAS-Islands).

**Figure 7:**
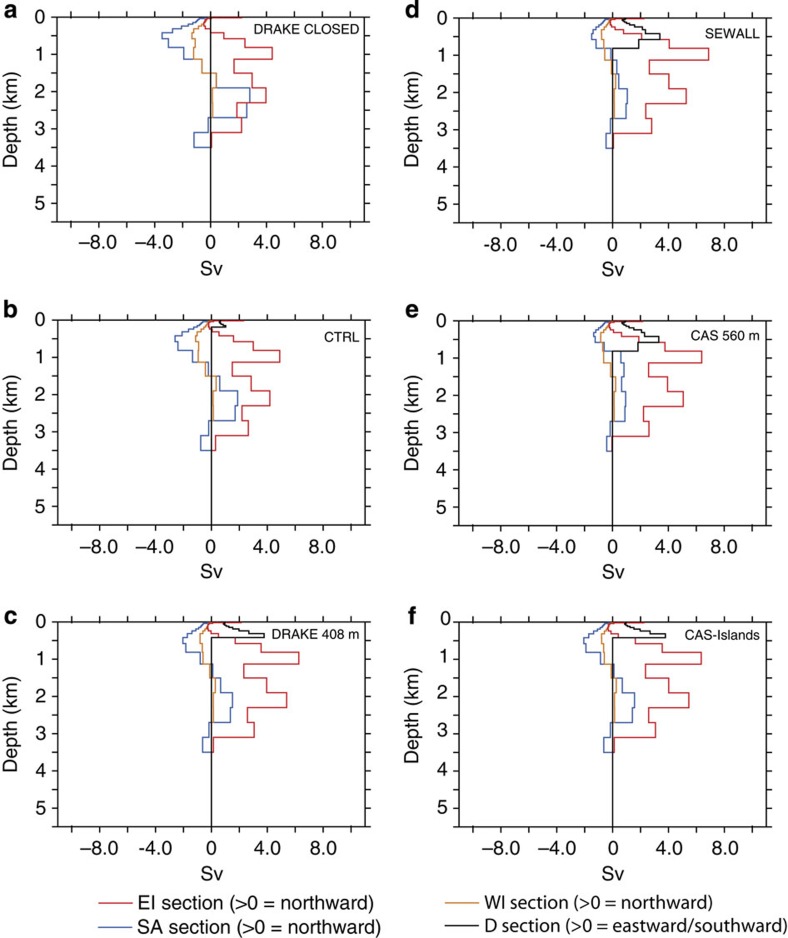
Water transport for the SA and Indian basins at 94 Ma. (**a**) Drake Passage closed (DRAKE CLOSED), (**b**) Drake Passage at 145 m (CTRL), (**c**) Drake Passage at 408 m (DRAKE408m), (**d**) Drake Passage as in Sewall *et al.*^9^ (SEWALL), (**e**) Caribbean gateway at 560 m (CAS560m) and (**f**) Continental plate across the Caribbean gateway (CAS-Islands).

**Figure 8:**
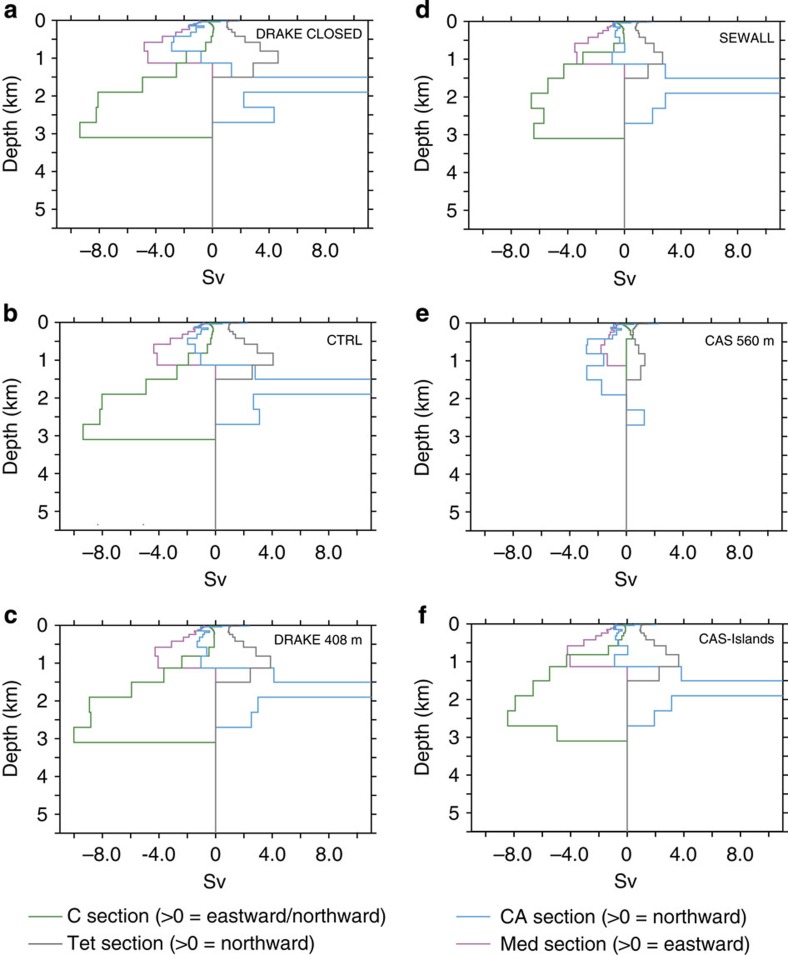
Water transport for the North Atlantic and the Tethys basins at 71 Ma. (**a**) Drake Passage closed (DRAKE CLOSED), (**b**) Drake Passage at 145 m (CTRL), (**c**) Drake Passage at 408 m (DRAKE408m), (**d**) Drake Passage as in Sewall *et al.*^9^ (SEWALL), (**e**) Caribbean gateway at 560 m (CAS560m) and (**f**) continental plate across the Caribbean gateway (CAS-Islands).

**Figure 9:**
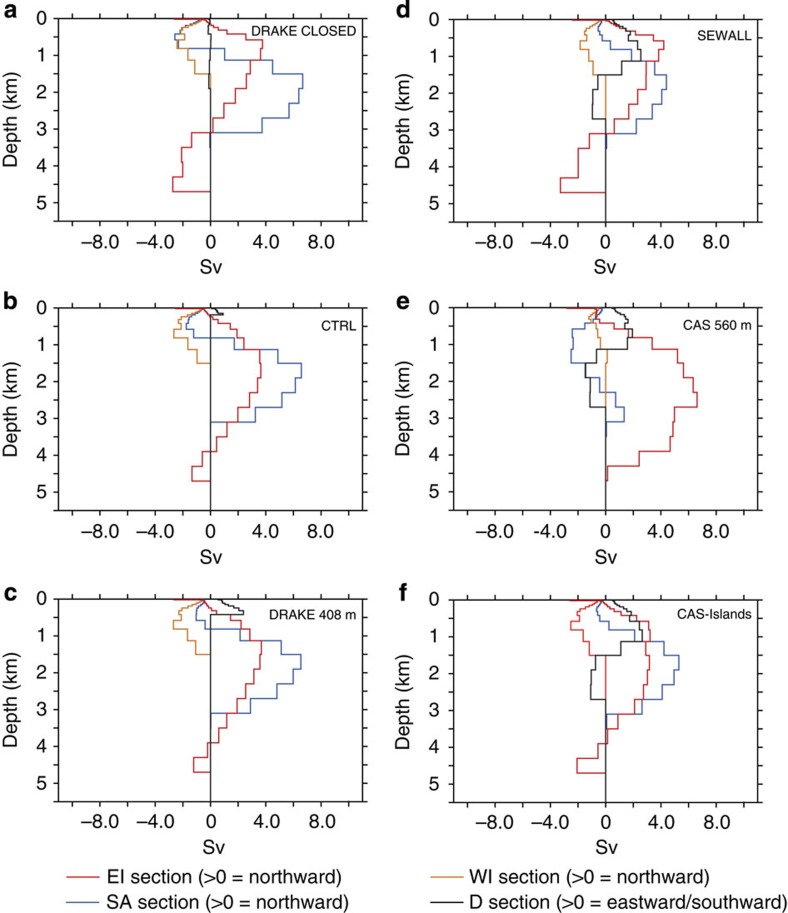
Water transport for the SA and Indian basins at 71 Ma. (**a**) Drake Passage closed (DRAKE CLOSED), (**b**) Drake Passage at 145 m (CTRL), (**c**) Drake Passage at 408 m (DRAKE408m), (**d**) Drake Passage as in Sewall *et al.*^9^ (SEWALL), (**e**) Caribbean gateway at 560 m (CAS560m) and (**f**) continental plate across the Caribbean gateway (CAS-Islands).

To test the impact of the configuration of the Drake Passage and of the Caribbean Seaway on oceanic circulation, we performed a series of sensitivity experiments. In a first set of simulations, we modified the Cenomanian/Turonian and the early Maastrichtian palaeogeographies of Sewall *et al.*^9^ using the same horizontal configuration but varying the depth of the Drake Passage, using the depth originally specified by Sewall *et al.*^9^, which is around 1,000 m (SEWALL simulations), a depth of 408 m (DRAKE408m simulations), a depth of 145 m (CTRL simulations) and a closed Drake Passage (DRAKE CLOSED simulation). In a second set of simulations, two runs for each palaeogeography were performed with a very shallow Caribbean Gateway, fixed at 560 m depth (CAS560m simulations) and a small continental plate across the Caribbean Seaway (Supplementary Fig. 8) to simulate the presence of islands (CAS-Islands simulation). The results of these experiments are presented in Figs 6, 7, 8, 9 and in Table 1.

Most features described above for the two land–sea masks in the CTRL runs remain the same independently of the depth of the Drake Passage (Figs 6, 7, 8, 9). These features are the inversion of the direction of deep-water flow across the Caribbean Seaway for depths >1,000 m and the increase in northward water fluxes at depth across the SA basin. Summed over the whole water column, water flows globally from the North Atlantic to the SA during the Cenomanian–Turonian and in the opposite direction during the early Maastrichtian. However, the depth of the Drake Passage substantially affects the intensity of these flows. The main changes occur when going from a closed passage to a depth of 408 m. Most notably, the deeper branch of the thermohaline circulation coming from the Weddell Sea decreases its volume transport from 5.8 to 3.7 Sv for the 94 Ma experiments and from 35.4 to 16.2 Sv for the 71 Ma experiments (Table 1). Nevertheless, the trend from a sluggish circulation to a more active circulation at depth in the South and CA with the early Maastrichtian palaeogeography holds independently of the configuration of the Drake Passage.

Sensitivity experiments assessing the effect of Caribbean Seaway depth on circulation yield contrasting results, especially for the early Maastrichtian simulations. A shallow gateway across the Caribbean Seaway (CAS560m simulations) completely modifies deep circulation and results in a shut down of thermohaline circulation in the SA (Figs 8 and 9). By contrast, the same configuration but for the 94 Ma experiments has a minor impact on the general pattern of oceanic circulation (Figs 6 and 7). Finally, experiments with a small continental plate across the Caribbean Seaway result in only minor changes in the modelled oceanic circulation for both palaeogeographies.

The circulation modelled with a shallow Caribbean Seaway (CAS560 m) for the Cenomanian/Turonian is less consistent with the *ɛ*_Nd_ , because a deep-water influx of radiogenic Pacific waters through the Caribbean Seaway as observed in the other simulations is in better agreement with the radiogenic composition of North Atlantic deep waters. In the early Maastrichtian, the weaker fluxes of surface and intermediate waters across the Tethys, Med and Caribbean Seaway modelled in the CAS560m experiment are also less consistent with an intense equatorial circumglobal current that has been suggested based on the large-scale deposition of phosphorites along the southern Tethyan margin^47^.

These additional sensitivity experiments reveal a major role of the circulation pattern across the Caribbean Seaway on the overall modelled thermohaline circulation and shed new light on the mechanisms driving the change in oceanic circulation observed between the simulations using the Cenomanian/Turonian versus early Maastrichtian palaeogeographies. The hydrological cycle changes described above contribute to the intensification of deep-water production in the southern Atlantic and Indian Oceans. Changes in land–sea configuration and the associated atmospheric feedbacks induce a saltier subtropical SA Ocean during the early Maastrichtian, which in turn results in a larger area of deep-water formation around the eastern Antarctic (Fig. 1). Indeed, the saltier the surface waters are, the denser and heavier they will be when they reach the southern high latitudes. An additional important factor appears to be the strong flow of Atlantic water into the Pacific at all depths during the Maastrichtian, observed in all experiments, except the CAS 560 m simulation. This flow creates a large divergence area in the CA, drawing in waters from the Med gateway and from the SA (Table 1). The transition from an estuarine circulation during the Cenonamian–Turonian to the one characterizing the early Maastrichtian as simulated here is probably the main mechanism explaining the shift from a sluggish to an active deep circulation. This transition occurs in response to continental drift and the subsequent modification of the geometry of the Caribbean Seaways. The sensitivity of oceanic circulation across the Caribbean Seaways has already been investigated many times and results from a complex interplay between local processes, such as wind-driven circulation and salinity contrast^45^, and remote processes such as the geometry of other gateways^46^,^48^.

## Discussion

Our simulations highlight an evolution from a sluggish circulation in the SA and CA using the Cenomanian/Turonian land–sea mask towards a much more active circulation in these basins with the early Maastrichtian land–sea mask. Alongside changes in the location of deep-water production areas at high latitudes, these features may have paved the way for a well-oxygenated deep ocean. The absence of large-scale anoxia in deep waters during abrupt warming events after the Late Cretaceous, such as the Paleocene–Eocene transition^49^ (∼55 Ma), supports this hypothesis. The experiments conducted here emphasize the potentially important role of land–sea configuration as a pre-conditioning factor that affects the ease with which OAEs can develop. In that sense, palaeogeography represents one of the required conditions for the occurrence of OAEs but would not be sufficient on its own to trigger these events, implying additional causal factors^50^,^51^. As a result of the palaeogeographic changes occurring during the Late Cretaceous, our results suggest that worldwide anoxic conditions in deep waters were more likely to develop as a result of the same triggering factor (for example, increased nutrient input into the oceans^2^,^3^,^4^) during the Cenomanian/Turonian compared with the early Maastrichtian. Two recent geochemical modelling studies have emphasized the need for a sluggish circulation in the Atlantic basin, to explain the large spatial extent of OAE-2 (ref. 52) and a substantial sensitivity of the onset of anoxia/euxinia to oceanic overturning^53^. Based on the latter study, we predict that in a sluggish state such as the one simulated at the Cenomanian/Turonian boundary with <5 Sv of deep water flowing northward in the SA, a 20–40% increase in nutrient input to the ocean could generate deep-water euxinia. In contrast, a 225% increase in nutrient input is required to provoke euxinia with the early Maastrichtian palaeogeography in which 25.8 Sv feed the deep-water flowing northward (Fig. 3c,d). Our results imply that thresholds for the ocean-climate system to shift towards a state of global anoxia in deep waters are likely to be much higher at present than during the Cenomanian/Turonian because of the strength of the modern thermohaline circulation.

In summary, this study provides a clear description of oceanic circulation changes occurring during the Late Cretaceous and is able to broadly explain available published *ɛ*_Nd_ data. Our work points to changes in continental configuration as the major driver for the depicted ocean circulation change, through a modification of the hydrological cycle over the SA basin and the development of a strong westward flow of water at all depths throughout the Caribbean Seaway, that both favour an intensification of deep-water production in the southern Atlantic and Indian region. We note that our knowledge of the geometry of Cretaceous oceanic gateways is contentious and a definitive history of ocean circulation during this time must await further constraints on the state of the various oceanic connections. We also speculate on the consequences of long-term oceanic circulation changes on the likelihood that the Earth System will be affected by an OAE. More explicit modelling including complex climate models and biogeochemistry will help reveal the extent to which the tectonic and climatic background state can influence the development of oceanic anoxic conditions.

## Methods

### Code availability

The code of the model FOAM is available on request by e-mail to the first author.

### Description of the model

The model experiments were completed using the FOAM, developed by Jacob^54^. This model combines a low spectral resolution R15 (48 × 40 grid) atmosphere model counting 18 altimetric levels with a highly efficient medium resolution (128 × 128 grid) ocean module composed by 24 bathymetric levels. FOAM successfully simulates many aspects of the present-day climate and compares well with other contemporary medium-resolution climate models. It was previously used to investigate numerous past climate changes, ranging from the Neoproterozoic glaciations to the onset of the ACC at the Eocene–Oligocene boundary^55^,^56^.

### Description of the experiments

The two sets of simulations (called hereafter CTRL94Ma and CTRL71Ma) developed for this study are based on existing palaeogeographic reconstructions for the Cenomanian/Turonian boundary and for the early Maastrichtian^9^ (Fig. 2). For the sake of simplicity, we choose to use 94 Ma though the accurate age of the boundary is 93.9 Ma^57^. Based on existing palaeogeographies^41^,^58^,^59^, the depth of the Drake Passage in the CTRL simulations has been restricted to a shallower depth (that is, 145 m) than in the original bathymetry in which the depth reached more than a thousand metres. Indeed, first evidences for the deepening of the Drake Passage correspond to the creation of oceanic crust and are generally dated between the Eocene and the Oligocene^60^,^61^. During the Late Cretaceous, pull-apart basins were formed between the Antarctica Peninsula and southern South America^62^, which represent the onset of the opening of the Scotia Sea. Therefore, the Drake Passage most probably formed a narrow and shallow marine domain at that time^63^. To account for existing uncertainties on the configuration of the Drake Passage and of the Caribbean Seaway, sensitivity experiments assessing the effect of the depth specified for these two marine gateways are also presented in the discussion. Data and model-based atmospheric CO_2_ level estimates for the Late Cretaceous are between two and eight times the pre-industrial atmospheric CO_2_ concentration (280 p.p.m.)^64^,^65^,^66^,^67^,^68^,^69^. Accordingly, three atmospheric CO_2_ concentrations were tested for the two palaeogeographies: 560, 1,120 and 2,240 p.p.m. The solar luminosity was specified as 99% of modern. Orbital parameters and other greenhouse gases were set to present-day values. River routing is specified using the Sewall *et al.* reconstruction.

The experiments were integrated for 2,000 years without flux corrections or deep ocean acceleration. During the last 100 years of model integration, there is no apparent drift in the upper ocean (between the surface and 300 m depth) and <0.1 °C per century change in globally averaged ocean temperature. All model results have been averaged over the last 50 model years.

## Additional information

**How to cite this article:** Donnadieu, Y. *et al.* A better-ventilated ocean triggered by Late Cretaceous changes in continental configuration. *Nat. Commun.* 7:10316 doi: 10.1038/ncomms10316 (2016).

## Supplementary Material

Supplementary Figures and References Supplementary Figures 1-8 and Supplementary References

Supplementary Data 1 Neodymium isotope composition of modern detrital material on continental margins. Database from Jeandel et al., (2007) with additional references included

Supplementary Data 2 Compilation of eNd(t) of intermediate to deep waters at ODP sites for the Cenomanian/Turonian boundary and for the early Maastrichtian

## Figures and Tables

**Table 1:** Water fluxes integrated over the water column for each section defined in Figure 1.

	**Drake**	**SA**	**WI**	**EI**	**C**	**CA**	**Med**	**Tet**
*94 Ma*								
DRAKE CLOSED	0	−15 (5.8)	−5	20	−2.3	−15.1	−17.4	17.3
**CTRL**	**7**	**−11.6 (4.2)**	**−6.4**	**25**	**−5.6**	**−11.7**	**−17.4**	**17.3**
DRAKE408m	16	−9 (3.6)	−5.2	30.2	−7.2	−9.2	−16.5	16.3
SEWALL	17.6	−7 (3.7)	−8.	32.6	−9	−7.	−16.	16
CAS560m	17.2	−4.6 (3.9)	−8.4	30.2	−16.2	−4.7	−21.1	21
CAS-Islands	16	−8.8 (3.8)	−4.5	29.3	−8.1	−8.9	−17	16.9
*71 Ma*								
DRAKE CLOSED	0	12.3 (35.4)	−9.5	21.8	−34.9	12.3	−22.5	21.7
**CTRL**	**6.25**	**14.9 (25.8)**	**−21.9**	**13.3**	**−39.5**	**14.9**	**−24.8**	**23.8**
DRAKE408m	12.4	18.7 (23.7)	−19.8	13.5	−41.1	18.7	−22.4	22.1
SEWALL	14.4	15.6 (16.2)	−15.3	14.1	−33.6	15.6	−18.1	18.6
CAS560m	10.1	−14 (1.1)	−9.8	33.8	2	−14	−12.1	11.6
CAS-Islands	14.6	17.7 (22.1)	−18.5	15.4	−41.1	17.7	−23.4	23.5

C, Caribbean Seaway; CA, Central Atlantic; CAS560m, Caribbean gateway at 560 m; D, Drake Passage; DRAKE408m, Drake Passage at 408 m; EI, East India; Med, Mediterranean; SA, South Atlantic; Tet, Tethys; WI, West India.

Additional numbers in SA column correspond to the deep flow going northward in the South Atlantic across the SA section. Numbers in bold put forward the main simulations discussed in the paper, that is, with a shallow Drake Passage at 145 m.
