# Supplementary material for: A better-ventilated ocean triggered by Late Cretaceous changes in continental configuration
Source: Nat Commun. 2016 Jan 18;7:10316. doi: 10.1038/ncomms10316 (PMC4735640; doi:10.1038/ncomms10316)
Supplement: Supplementary Figures and References — Supplementary Figures 1-8 and Supplementary References [file ncomms10316-s1.pdf]

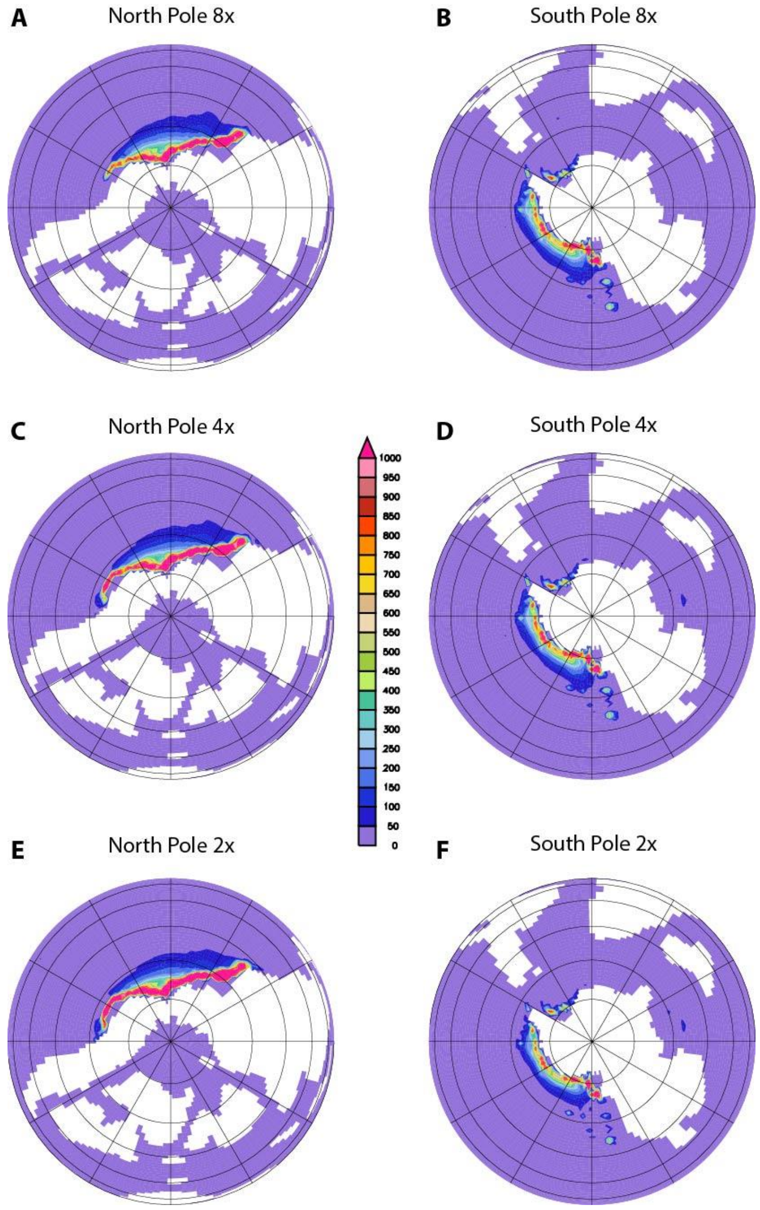

Supplementary Figure 1. Annual distribution of convective adjustments across the water column for the 95 Ma runs at various CO_2 levels (8x, 4x, and 2x the pre-industrial CO_2 atmospheric level of 280 ppm). The shading signifies the number of times the water column have undergone convective mixing summed over a year. Regions experiencing a large occurrence of convective adjustments are interpreted to represent site of intermediate and deep-water formation.

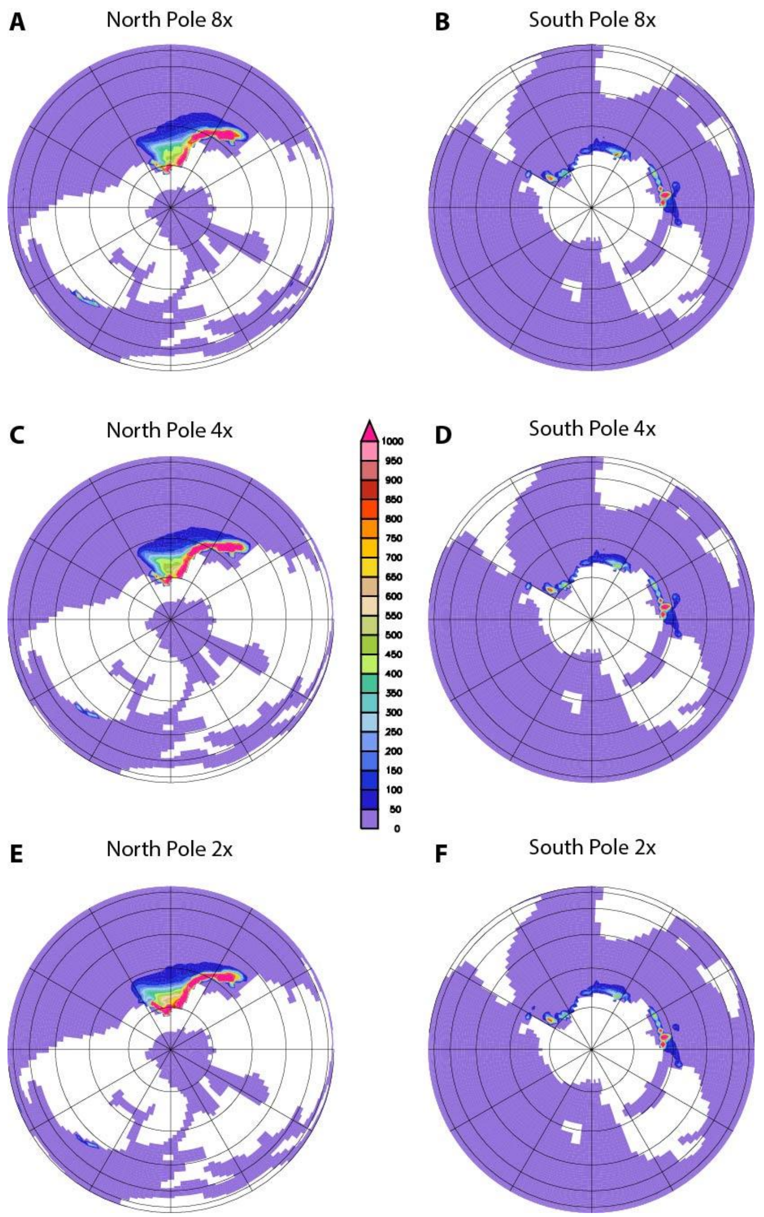

Supplementary Figure 2. Annual distribution of convective adjustments across the water column for the 70 Ma runs at various CO_2 levels (8x, 4x, and 2x the pre-industrial CO_2 atmospheric level of 280 ppm). The shading signifies the number of times the water column have undergone convective mixing summed over a year. Regions experiencing a large occurrence of convective adjustments are interpreted to represent site of intermediate and deep-water formation.

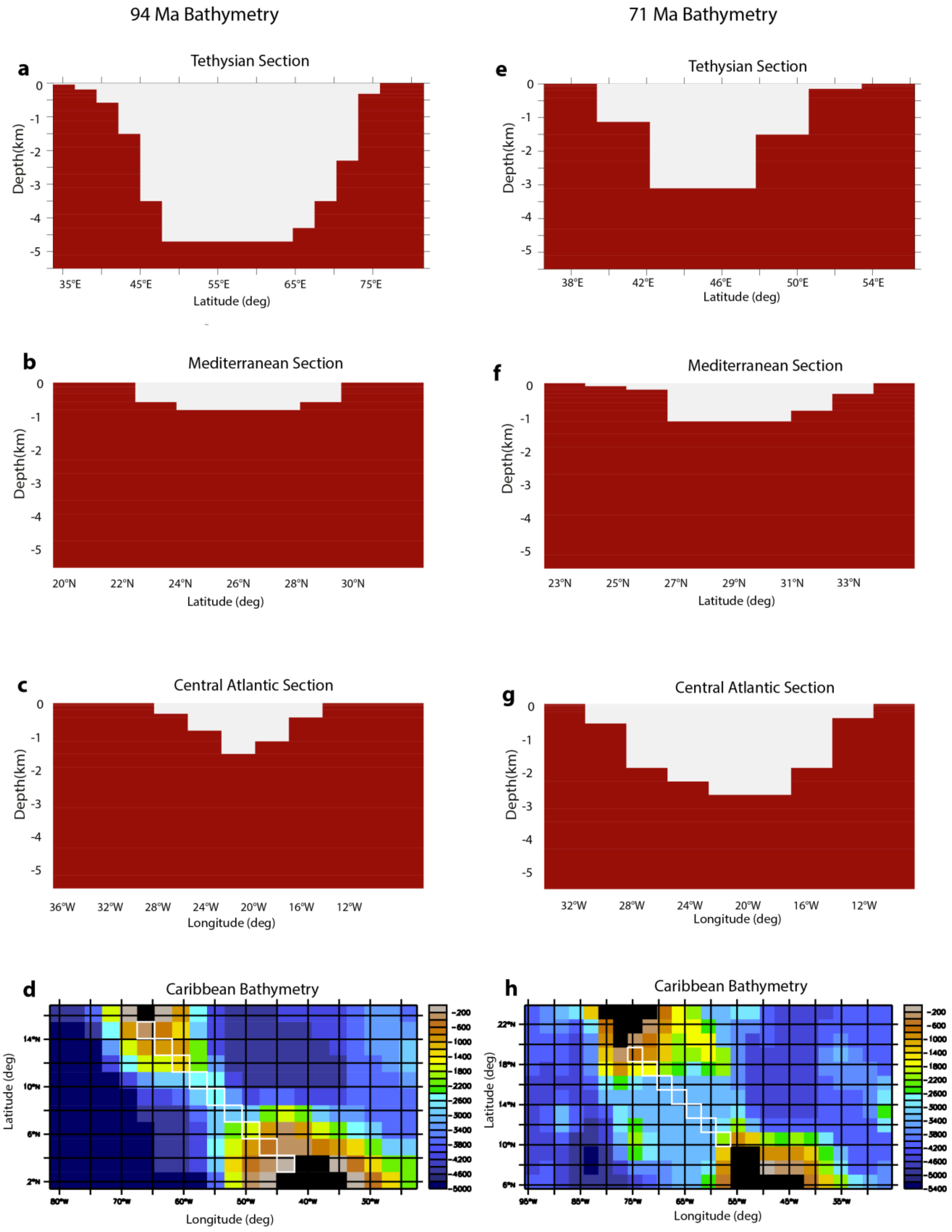

Supplementary Figure 3. Details of the bathymetry for each section shown in figure 1 for the North Atlantic and Tethys basins.

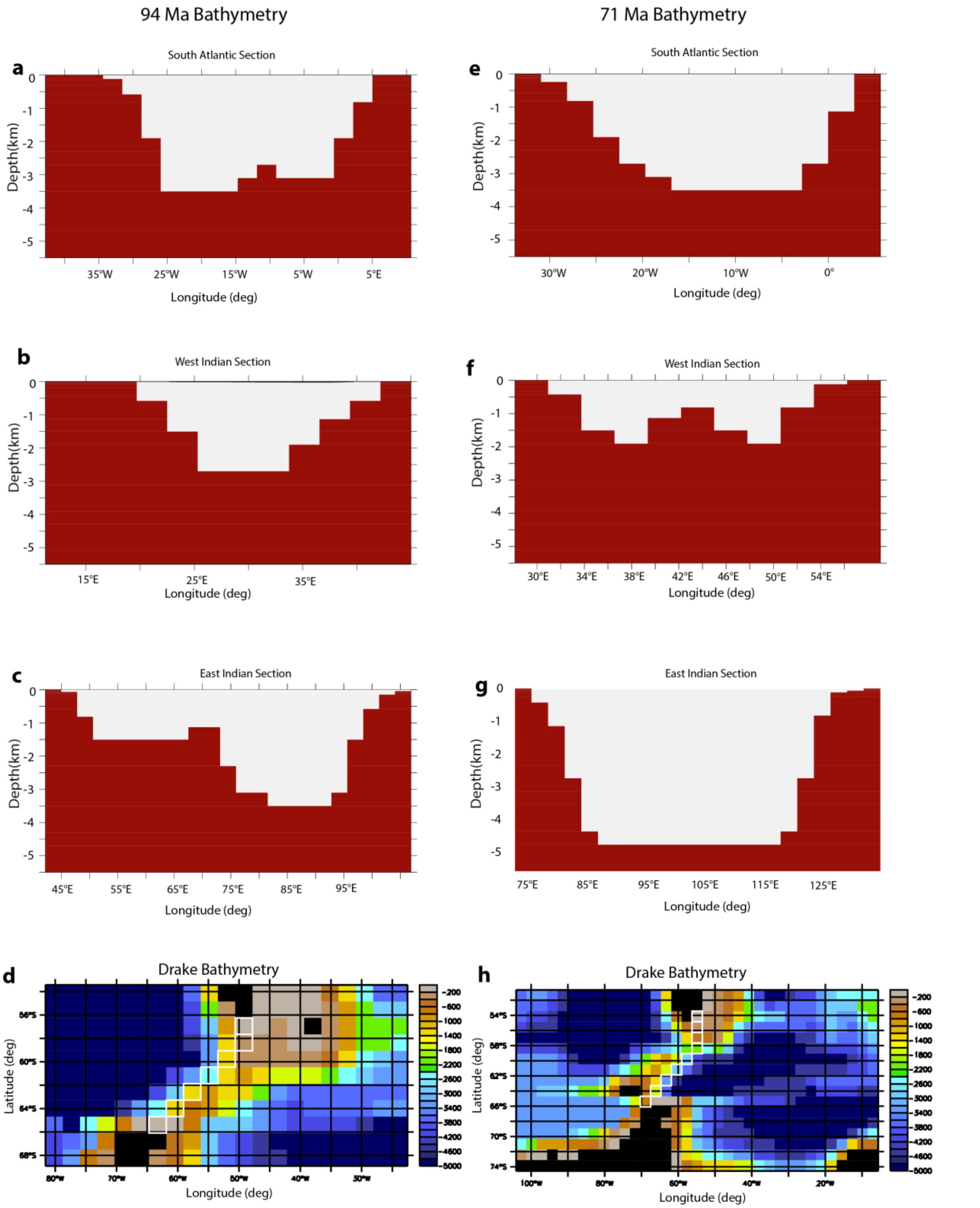

Supplementary Figure 4. Details of the bathymetry for each section shown in figure 1 for the South Atlantic and Indian basins.

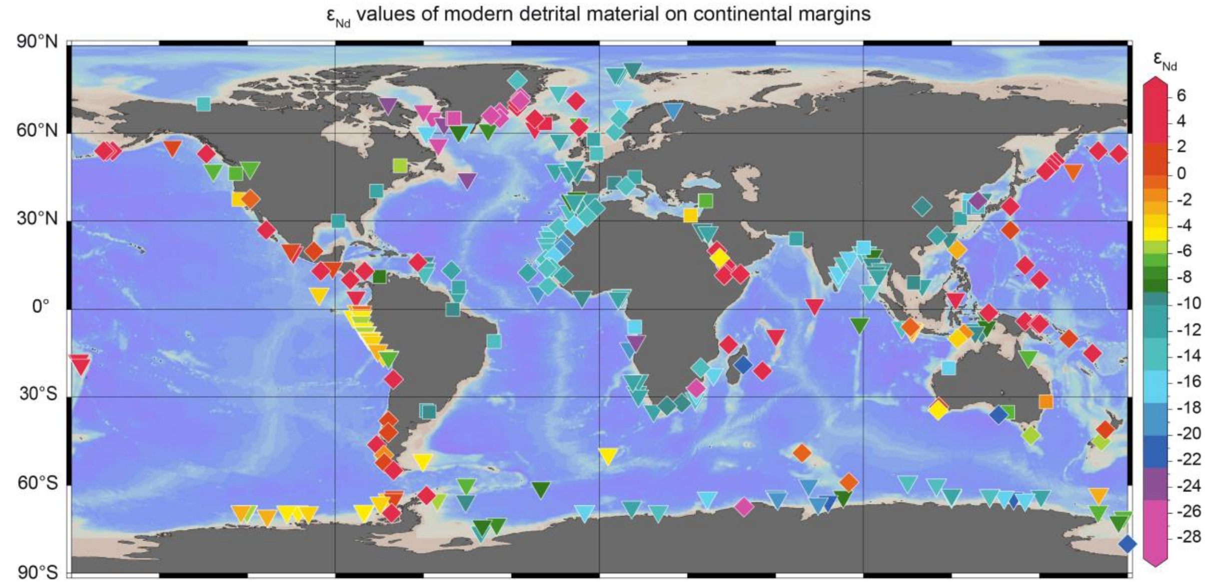

Supplementary Figure 5. Neodymium isotope values of modern sediments on continental margins. Diamonds represents lithogenic inputs, squares represent river loads, and inverse triangles represent top core sediments. Corresponding database[1-7] are in Supplementary Data 1.

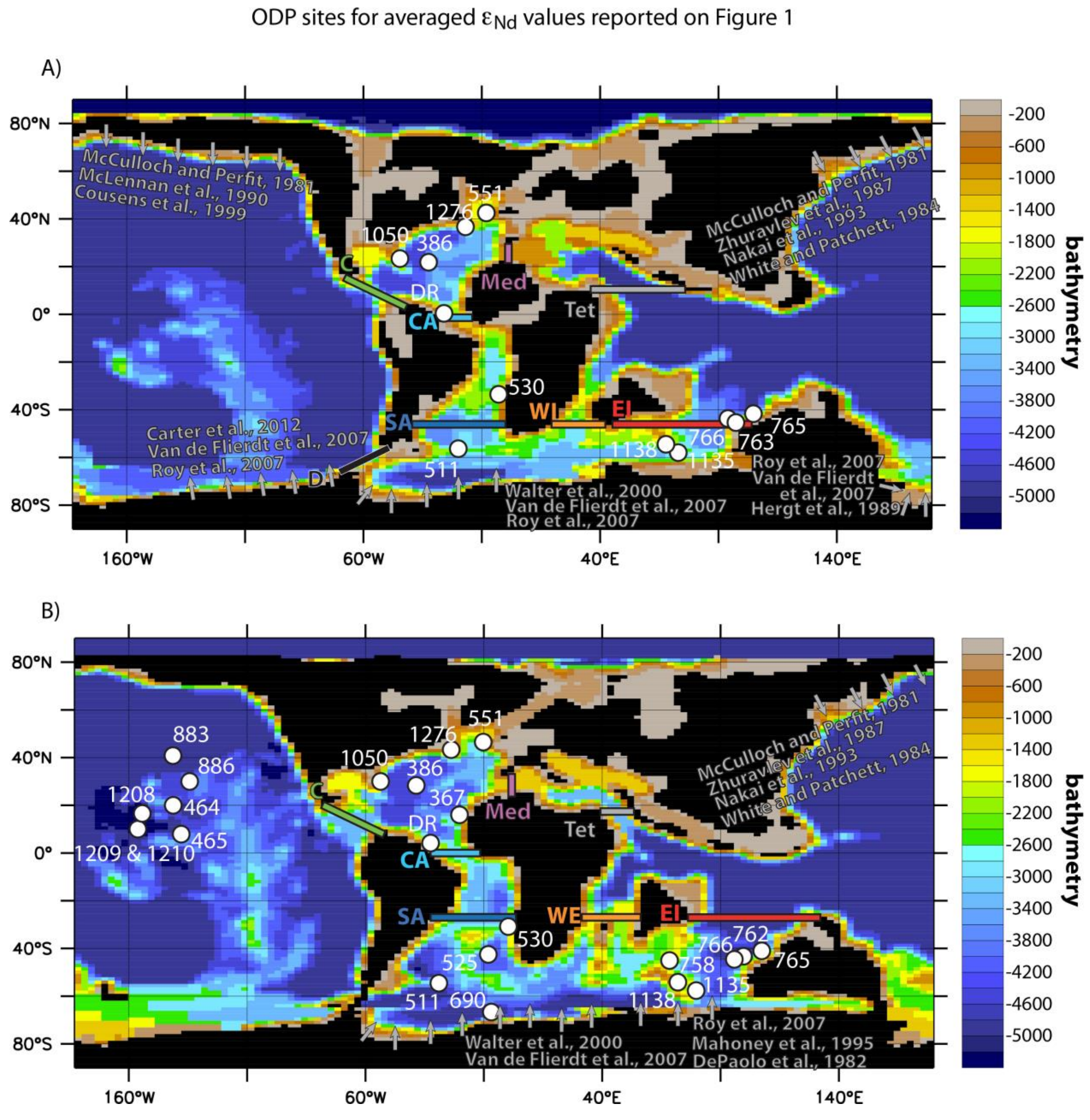

Supplementary Figure 6. Location of ODP sites (in white) for averaged neodymium isotope values[8-19] of seawater reported on figure 1 and on Supplementary Data 2. A) Paleomap for the Cenomanian/Turonian boundary). B) Paleomap for the early Maastrichtian. References[2,5,6,20-29] for the range of modern detrital ε_{Nd} values reported on Figure 1 are also highlighted here in grey. Geological timescales[30-34] used are visible on Supplementary Data 2.

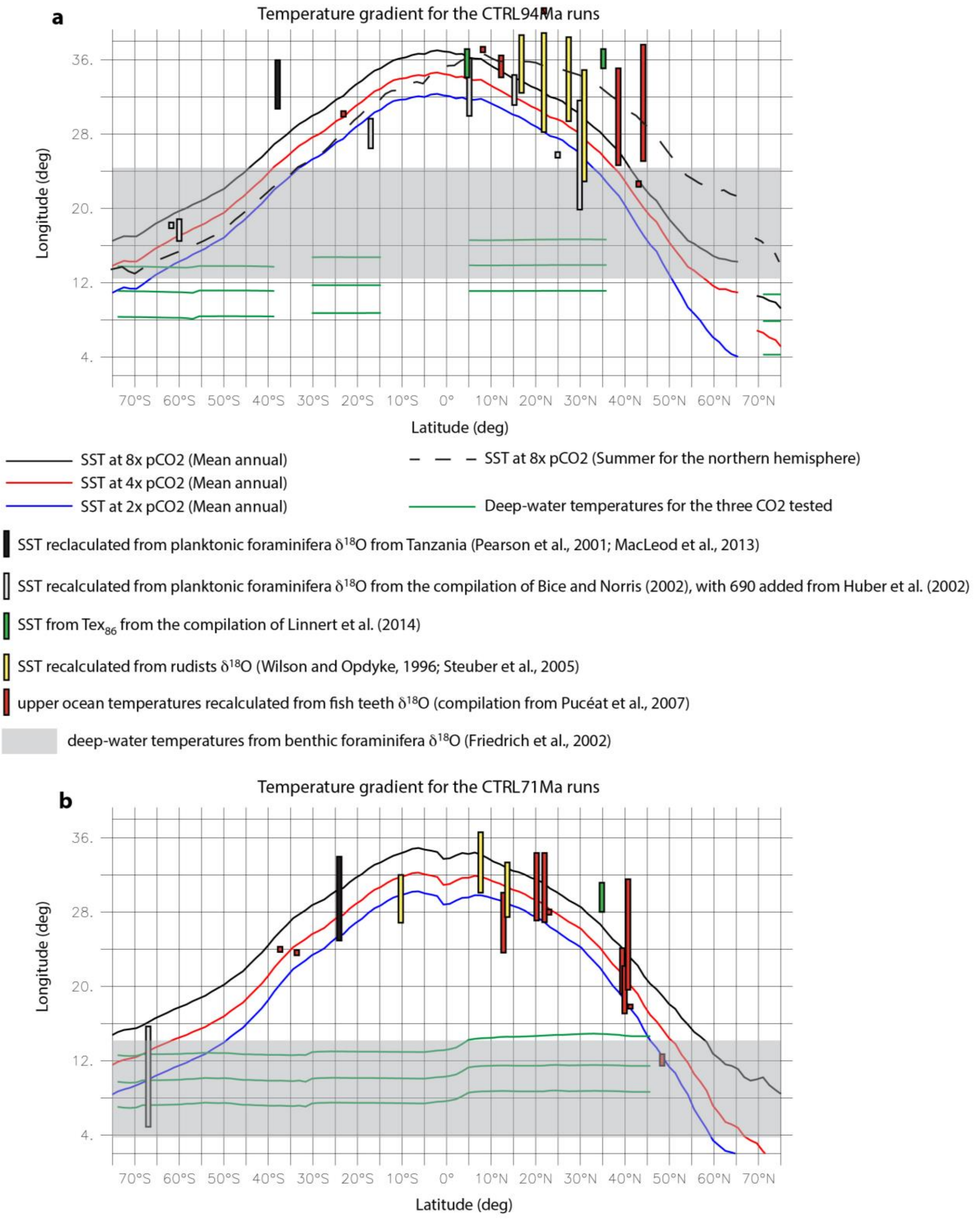

Supplementary Figure 7. Comparison between our climate simulations and the latitudinal thermal gradient based on data reconstructions. Thermal gradients of the simulations have been calculated by doing an average over the longitudes including the South Atlantic sector and the Tethysian area. The SST and upper ocean temperature ranges from $\delta^{18}O$ data presented on this figure are recalculated from (1) planktonic foraminifera $\delta^{18}O$ using the equation 1 of Bemis et al. (1998), (2) rudist $\delta^{18}O$ using the

equation of Anderson and Arthur (1983) for calcitic rudists, (3) apatite fish tooth $\delta^{18}O$ using the equation of Pucéat et al. (2010) for all data from the compilation of Pucéat et al. (2007) except for the data from Kolodny and Raab (1988) and Kolodny and Luz (1991) that have been calculated using the equation of Kolodny et al. (1983). A $\delta^{18}O$ of seawater of -1‰ SMOW (or -1,27‰ PDB) has been used to account for the absence of well-developed ice-sheets[46] and applying an additional correction for local variations of surface water $\delta^{18}O$ with latitude using the equation of Zachos (1994). SST from Tex86 data are taken from Linnert et al. (2014). SST from aragonitic rudist from Wison and Opdyke (1996) have not been recalculated and are as in the original publication. Deep-sea temperatures range is from the compilation of benthic foraminifera $\delta^{18}O$ as in the original publication[50].

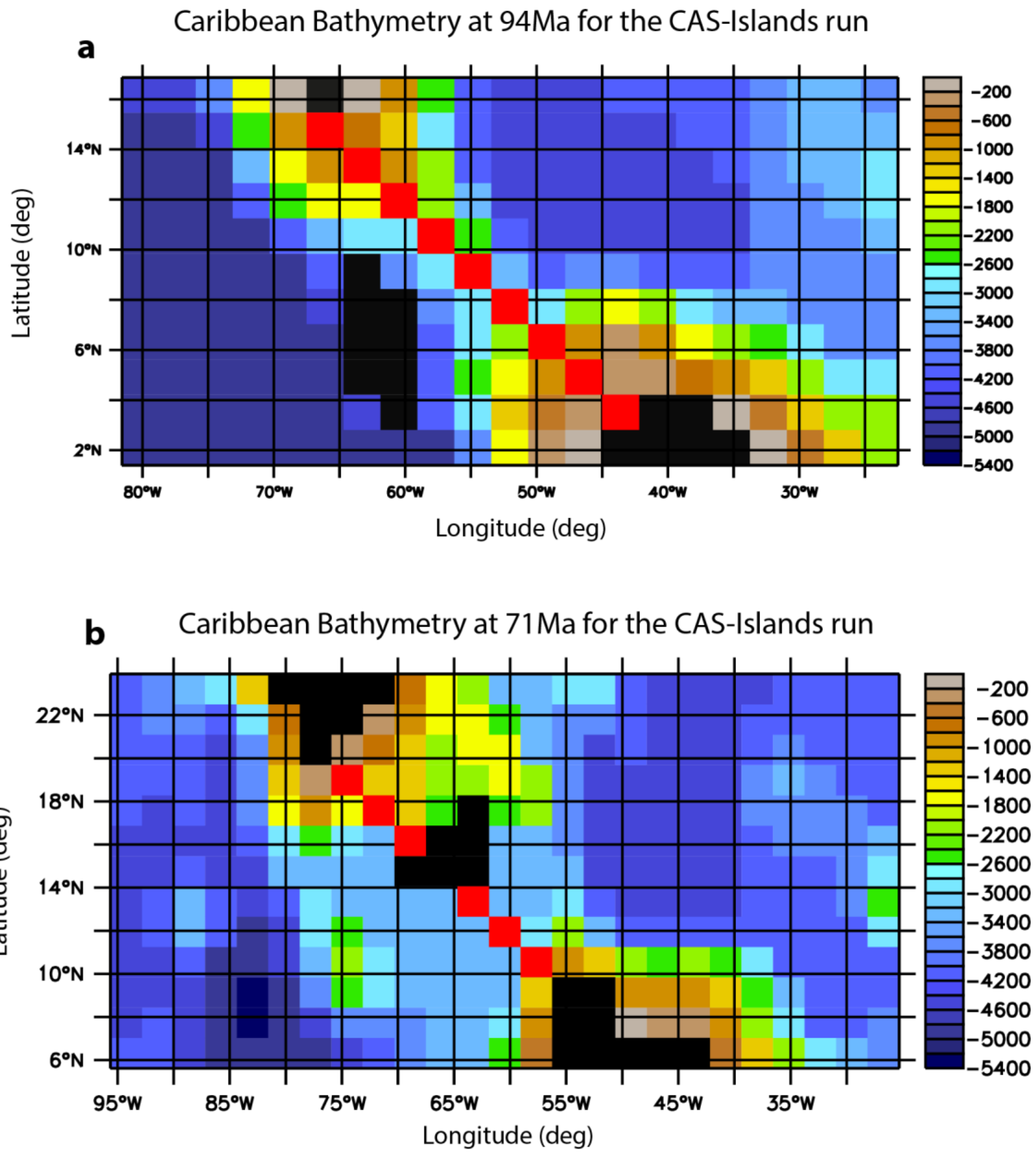

Supplementary Figure 8. Modified bathymetry across the Caribbean Seaway to account for the possibilty of small continental plates as used in the experiments CAS-Islands (see main text for details).

Supplementary References

1 Bayon, G. *et al.* Evidence for intense REE scavenging at cold seeps from the Niger Delta margin. *Earth and Planetary Science Letters* **312**, 443-452 (2011).

2 Carter, P., Vance, D., Hillenbrand, C. D., Smith, J. A. & Shoosmith, D. R. The neodymium isotopic composition of water masses in the eastern Pacific sector of the Southern Ocean. *Geochemica et Cosmochimica Acta* **79**, 41-59 (2012).

3 Ehlert, C., Grasse, P. & Frank, M. Changes in silicate utilisation and upwelling intensity off Peru since the Last Glacial Maximum – insights from silicon and neodymium isotopes. *Quaternary Science Reviews* **72**, 18-35 (2013).

4 Jeandel, C., Arsouze, T., Lacan, F., Téchiné, P. & Dutay, J.-C. Isotopic Nd compositions and concentrations of the lithogenic inputs into the ocean: A compilation, with an emphasis on the margins. *Chemical Geology* **239**, 156-164 (2007).

5 Roy, M., van de Flierdt, T., Hemming, S. R. & Goldstein, S. L. 40Ar/39Ar ages of hornblende grains and bulk Sm/Nd isotopes of circum-Antarctic glacio-marine sediments: Implications for sediment provenance in the southern ocean. *Chemical Geology* **244**, 507-519 (2007).

6 van de Flierdt, T. *et al.* Global neodymium-hafnium isotope systematics - revisited. *Earth and Planetary Science Letters* **259**, 432-441 (2007).

7 Werner, K., Frank, M., Teschner, C., Müller, J. & Spielhagen, R. F. Neoglacial change in deep water exchange and increase of sea-ice transport through eastern Fram Strait: evidence from radiogenic isotopes. *Quaternary Science Reviews* **72**, 1-18 (2013).

8 Frank, T. D. *et al.* The Maastrichtian record from a depth transect on Shatski Rise (northwest Pacific): a tropical perspective on global ecological and oceanographic changes. *Paleoceanography* **20**, 1-14 (2005).

9 Hague, A. M. *et al.* Convection of North Pacific deep water during the early Cenozoic. *Geology* **40**, 527-530, doi:10.1130/g32886.1 (2012).

10 Le Houedec, S., Meynadier, L., Cogné, J. P., Allègre, C. J. & Gourlan, A. T. Oceanwide imprint of large tectonic and oceanic events on seawater Nd isotope composition in the Indian Ocean from 90 to 40 Ma. *Geochemistry Geophysics Geosystems* **13**, doi:10.1029/2011GC003963 (2012).

11 MacLeod, K. G., Londono, C. I., Martin, E. E., Berrocoso, A. J. & Basak, C. Changes in North Atlantic circulation at the end of the Cretaceous greenhouse interval. *Nature Geoscience* **4**, 779-782, doi:Doi 10.1038/Ngeo1284 (2011).

12 MacLeod, K. G., Martin, E. E. & Blair, S. W. Nd isotopic excursion across Cretaceous ocean anoxic event 2 (Cenomanian-Turonian) in the tropical North Atlantic. *Geology* **36**, 811-814, doi:Doi 10.1130/G24999a.1 (2008).

13 Martin, E. E. *et al.* Extraction of Nd isotopes from bulk deep sea sediments for paleoceanographic studies on Cenozoic time scales. *Chemical Geology* **289**, 414–431 (2010).

14 Martin, E. E., MacLeod, K. G., Berrocoso, A. J. & Bourbon, E. Water mass circulation on Demerara Rise during the Late Cretaceous based on Nd isotopes. *Earth and Planetary Science Letters* **327**, 111-120, doi:Doi 10.1016/J.Epsl.2012.01.037 (2012).

15 Murphy, D. P. & Thomas, D. J. Cretaceous deep-water formation in the Indian sector of the Southern Ocean. *Paleoceanography* **27**, PA1211, doi:10.1029/2011pa002198 (2012).
16 Robinson, S. A., Murphy, D. P., Vance, D. & Thomas, D. J. Formation of "Southern Component Water" in the Late Cretaceous: Evidence from Nd-isotopes. *Geology* **38**, 871-874, doi:Doi 10.1130/G31165.1 (2010).
17 Robinson, S. A. & Vance, D. Widespread and synchronous change in deep-ocean circulation in the North and South Atlantic during the Late Cretaceous. *Paleoceanography* **27**, doi:10.1029/2011pa002240 (2012).
18 Thomas, D. J. Evidence for deep-water production in the North Pacific Ocean during the early Cenozoic warm interval. *Nature* **430**, 65-68 (2004).
19 Voigt, S. *et al.* Tectonically restricted deep-ocean circulation at the end of the Cretaceous greenhouse. *Earth and Planetary Science Letters* **369-370**, 169-177 (2013).
20 Cousens, B. L., Dostal, J. & Hamilton, T. S. A near-ridge origin for seamounts at the southern terminus of the Pratt-Welker Seamount Chain, northeast Pacific Ocean. *Can J Earth Sci* **36**, 1021-1031 (1999).
21 Depaolo, D. J., Manton, W. I., Grew, E. S. & Halpern, M. Sm–Nd, Rb–Sr and U–Th–Pb systematics of granulite facies rocks from Fyfe Hills, Enderby Land, Antarctica. *Nature* **298**, 614-618 (1984).
22 Hergt, J. M., Chappell, B. W., Mc Culloch, M. T., McDougall, I. & Chivas, A. R. Geochemical and Isotopic Constraints on the Origin of the Jurassic Dolerites of Tasmania. *Journal of Petrology* **30**, 841-883 (1989).
23 Mahoney, J. J. *et al.* Geochemical characteristics of lavas from Broken Ridge, the Naturaliste Plateau and southernmost Kerguelen Plateau: Cretaceous plateau volcanism in the southeast Indian Ocean. *Chemical Geology* **120**, 315-345 (1995).
24 Mc Culloch, M. T. & Perfit, M. R. 143Nd/144Nd, 87Sr/86Sr and trace element constraints on the petrogenesis of Aleutian island arc magmas. *Earth and Planetary Science Letters* **56**, 167-179 (1981).
25 McLennan, S. M., Taylor, S. R., Mc Culloch, M. T. & Maynard, J. B. Geochemical and Nd/Sr isotopic composition of deep-sea turbidites: Crustal evolution and plate tectonic associations. *Geochemica et Cosmochimica Acta* **54**, 2015-2050 (1990).
26 Nakai, S., Halliday, A. N. & Rea, D. K. Provenance of dust in the Pacific Ocean. *Earth and Planetary Science Letters* **119**, 143-157 (1993).
27 Walter, H. J., Hegner, E., Diekman, B., Kuhn, G. & Loeff, M. M. R. V. D. Provenance and transport of terrigenous sediment in the south Atlantic Ocean and their relations to glacial and interglacial cycles: Nd and Sr isotopic evidence. . *Geochemica et Cosmochimica Acta* **64**, 3813-3827 (2000).
28 White, W. M. & Patchett, J. Hf-Nd-Sr isotopes and incompatible element abundances in island arcs: implications for magma origins and crust-mantle evolution. *Earth and Planetary Science Letters* **67**, 167-185 (1984).
29 Zhuravlev, D. Z., Tsvetkov, A. A., Zhuravlev, A. Z., Gladkov, N. G. & Chernyshev, I. V. 143Nd/144Nd and 87Sr/86Sr ratios in recent magmatic rocks of the Kurile island arc. *Chemical Geology* **66**, 227-243 (1987).
30 Berggren, W. A., Kent, D. V., Swisher, C. C. & Aubry, M.-P. *A revised geochronology and chronostratigraphy*. Vol. 54 129-212 (1995).

31 Cande, S. C. & Kent, D. V. R. c. o. t. g. p. t. f. t. l. C. a. C. J. G. R., 100:6093-6095. Revised calibration of the geomagnetic polarity timescale for the late Cretaceous and Cenozoic. *Journal of Geophysical Research* **100**, 6093-6095 (1995).
32 Gradstein, F. M. *et al. A Triassic, Jurassic and Cretaceous time scale*. Vol. 54 95-126 (1995).
33 Gradstein, F. M., Ogg, J. G. & Smith, A. G. *A Geologic Time Scale* (Cambridge University Press, 2004).
34 van Hinte, J. E. A Cretaceous time scale. *American Association of Petroleum Geology Bulletin* **60**, 498-516 (1976).
35 Hubert, B. T., Hodell, D. A. & Hamilton, C. P. Middle-Late Cretaceous climate of the southern high latitudes: Stable isotopic evidence for minimal equator-to-pole thermal gradients. *Geological Society of America Bulletin* **107**, 1164-1191 (1995).
36 MacLeod, K. G., Jiménez Berrocoso, A., Huber, B. T. & Wendler, I. A stable and hot Turonian without glacial d18O excursions is indicated by exquisitely preserved Tanzanian foraminifera. *Geology* **41**, 1083-1086 (2013).
37 Pearson, P. N. *et al.* Warm tropical sea surface temperatures in the Late Cretaceous and Eocene epochs. *Nature* **413**, 481-487 (2001).
38 Bice, K. L. & Norris, R. D. Possible atmospheric CO2 extrems of the Middle Cretaceous (late Albian-Turonian). *Paleoceanography* **17**, 1070, doi:1010.1029/2002PA000778 (2002).
39 Bemis, B. E., Spero, H. J., Bijma, J. & Lea, D. W. Reevaluation of the oxygen isotopic composition of planktonic foraminifera: experimental results and revised paleotemperature equations. *Paleoceanography* **13**, 150-160 (1998).
40 Anderson, T. F. & Arthur, M. A. in *Stable Isotopes in Sedimentary Geology Paleontologist and Mineralogists Short Course 10* (eds M. Arthur *et al.*) 1-151 (1983).
41 Pucéat, E. *et al.* Revised phosphate-water fractionation equation reassessing paleotemperatures derived from biogenic apatite. *Earth and Planetary Science Letters* **298**, 136-142 (2011).
42 Puceat, E. *et al.* Fish tooth delta 18O revising Late Cretaceous meridional upper ocean water temperature gradients. *Geology* **35**, 107-110 (2007).
43 Kolodny, Y. & Raab, M. Oxygen isotopes in phosphatic fish remains from Israel: Paleothermometry of tropical Cretaceous and Tertiary shelf waters. *Palaeogeography Palaeoclimatology Palaeoecology* **64**, 59-67 (1988).
44 Kolodny, Y. & Luz, B. Oxygen isotopes in phosphates of fossil fish: Devonian to recent. *Special Publication of the Geochemical Society* **3**, 105-119 (1991).
45 Kolodny, Y., Luz, B. & Navon, O. Oxygen Isotope Variations in Phosphate of Biogenic Apatites .1. Fish Bone Apatite - Rechecking the Rules of the Game. *Earth and Planetary Science Letters* **64**, 398-404 (1983).
46 Shackleton, N. J. & Kennett, J. P. Paleotemperature history of the Cenozoic and the initiation of Antarctic glaciation: oxygen and carbon analyses in DSDP sites 277, 279 and 281. 743-756 (Washington, D.C., 1975).
47 Zachos, J. & Stott, L. D. Evolution of early Cenozoic marine temperatures. *Paleoceanography* **9**, 353-387 (1994).
48 Linnert, C. *et al.* Evidence for global cooling in the Late Cretaceous. *Nature Communications* **5**, doi:10.1038/ncomms5194 (2014).

49 Wilson, P. A. & Opdyke, B. N. Equatorial sea-surface temperatures for the Maastrichtian revealed through remarkable preservation of metastable carbonate. *Geology* **24**, 555-558 (1996).
50 Friedrich, O., Norris, R. D. & Erbacher, J. Evolution of middle to Late Cretaceous oceans--A 55 m.y. record of Earth's temperature and carbon cycle. *Geology* **40**, 107-110, doi:10.1130/g32701.1 (2011).
